# Molecular Biomarkers of Brain and Spinal Cord Astrocytomas

**DOI:** 10.32607/20758251-2019-11-2-17-27

**Published:** 2019

**Authors:** N. A. Konovalov, D. S. Asyutin, E. G. Shayhaev, S. V. Kaprovoy, S. Yu. Timonin

**Affiliations:** National Medical Research Center of Neurosurgery, Ministry of Health of the Russian Federation Acad. N.N. Burdenko, 4th Tverskaya-Yamskaya Str. 16, Moscow, 125047, Russia; FGBU Russian Research Center for X-ray Radiology of the Ministry of Health of the Russian Federation Profsouznaya Str. 86, Moscow, 117485, Russia

**Keywords:** spinal cord astrocytoma, glioblastoma, mutations, molecular markers, diagnosis, mechanisms of neoplastic transformation, prognostic value

## Abstract

Spinal cord astrocytomas are rare diseases of the central nervous system. The localization of these tumors and their infiltrative growth complicate their surgical resection, increase the risk of postoperative complications, and require more careful use of radio- and chemotherapy. The information on the genetic mutations associated with the onset and development of astrocytomas provides a more accurate neoplasm diagnosis and classification. In some cases, it also allows one to determine the optimal methods for treating the neoplasm, as well as to predict the treatment outcomes and the risks of relapse. To date, a number of molecular markers that are associated with brain astrocytomas and possess prognostic value have been identified and described. Due to the significantly lower incidence of spinal cord astrocytomas, the data on similar markers are much more sparse and are presented with a lesser degree of systematization. However, due to the retrospective studies of clinical material that have been actively conducted abroad in recent years, the formation of statistically significant genetic landscapes for various types of tumors, including intradural spinal cord tumors, has begun. In this regard, the purpose of this review is to analyze and systematize the information on the most significant genetic mutations associated with various types of astrocytomas, as well as discuss the prospects for using the corresponding molecular markers for diagnostic and prognostic purposes.

## INTRODUCTION

Primary tumors of the spinal cord are rare diseases; they comprise only 2%–4% of all tumors of the central nervous system (CNS) [1, 2]. Symptoms associated with the development of such tumors can vary greatly depending on the tumor type and localization and include pain, autonomic, motor and sensory impairments, as well as dysfunction of pelvic organs [3]. Without treatment, they can lead to serious CNS dysfunction and patient death.

**Fig. 1:**
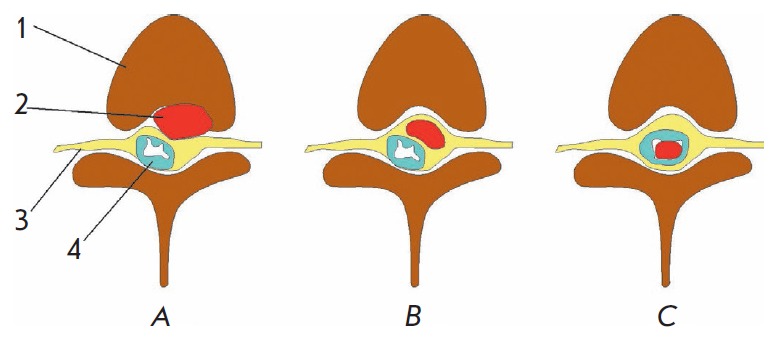
Types of spinal cord tumors: extradural extramedullary (*A*), intradural extramedullary (*B*), and intradural intramedullary (*C) *tumors. *1 *– vertebral body, 2 – tumor, *3 *– dura mater, and *4 *– spinal cord

Historically, there have been three main groups of spinal cord tumors: extradural extramedullary, intradural extramedullary, and intramedullary lesions (*Fig. 1*). The latter group (intramedullary spinal cord tumors, IMSCTs) is the rarest type of CNS neoplasms (5%–10% of all primary spinal cord neoplasms) [4, 5].

**Fig. 2:**
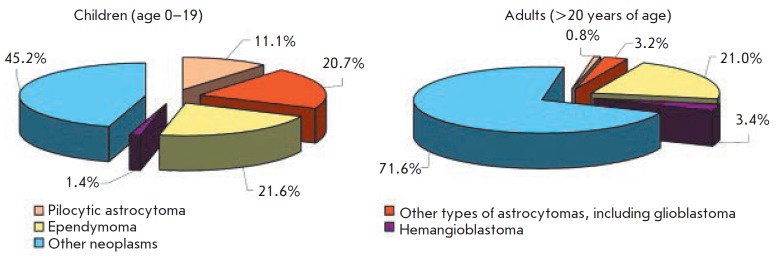
incidence of intradural intramedullary primary spinal cord tumors in children under the age of 19 years (*n *= 1,238) and adult patients (*n *= 14,822) according to the U.S. Central Brain Tumor Registry (CBTRUS) data report for 2007–2011. The data are presented according to [15] (with modifications)

The most frequent variants of IMSCTs are ependymomas and astrocytomas, which in total comprise about 90% (60% and 30%, respectively) of all IMSCT cases diagnosed in adults, while the remaining 10% include hemangioblastomas and metastatic tumors [6, 7]. On the contrary, in children under 10 years of age, astrocytomas are usually more common than ependymomas (*Fig. 2*) [8].

Astrocytomas develop from astrocytes, i.e., cells of the glial tissue. Therefore, they belong to the class of glial tumors. According to the WHO classification, there are four types of astrocytomas [9]. Pilocytic astrocytoma (PA, grade I) is a benign, slowly growing tumor separated from healthy tissues, which includes parallel hair-like bundles of glial fibers. It occurs mainly in patients under the age of 20; the 10-year survival rate exceeds 90% [10, 11]. Diffuse or low-grade astrocytoma (grade II) is an infiltrative tumor with no clear boundaries characterized by slow invasive growth, which gradually progresses to an anaplastic form. Anaplastic astrocytoma (grade III) is an infiltrative malignant tumor of heterogeneous structure which can either arise independently or develop from tumors with a lower grade of malignancy. Anaplastic astrocytoma is characterized by rapid progression and a steady decrease in cell differentiation to atypical glioblastoma. Glioblastoma (grade IV) is a tumor characterized by a high degree of malignancy and rapid infiltrative growth. Glioblastomas can occur *de novo *or develop from tumors of lower grades; they are diagnosed mainly in older patients [12].

In most cases, the detected astrocytomas belong to the grade I or II (85–90%), while the most malignant grades III and IV astrocytomas account for about 10–15% of all cases, with the frequency of a diagnosis of glioblastoma being only 0.2–1.5% [4]. In general, the incidence of primary spinal cord astrocytomas (SCA) is about 2.5 per 100,000 people per year [4]. Clinical manifestations of SCA largely depend on its localization and malignancy degree and most often include pain (~ 70%), sensory disorders (~ 65%), and motor function impairments (~ 50%) [13].

The understanding of the molecular biology of intracranial astrocytomas has significantly expanded over the past 10 years. In particular, some molecular parameters have been included to the WHO classification of CNS tumors (2016) [14]. Meanwhile, the research into the mechanisms of emergence and progression of malignant spinal cord astrocytomas, as well as the development of effective therapy methods, is progressing rather slowly, while the number of publications devoted to this type of tumors is very small compared to the data accumulated on intracranial astrocytomas. The primary reason is the rare incidence of this type of tumors and, therefore, the challenges associated with obtaining a statistically significant number of samples for analysis. In addition, the heterogeneity of the clinical presentation and various treatment strategies make it difficult to conduct a randomized study under standardized conditions [15]. Finally, the small size, localization of these tumors in the parenchyma, and the degree of their infiltration into the surrounding healthy tissues, which significantly increases the risk of complications associated with their surgical resection, make it very difficult to obtain enough tissue material for research. Meanwhile, the data on genetic changes in SCA cells provide information on the pathophysiological origin of the neoplasm and possible tumor markers; they can also allow one to determine the therapy option, predict the patient’s condition and the risk of recurrence [16]. Genetic studies on intracranial astrocytomas have laid the foundation for identifying the candidate genes responsible for the development of SCA, despite the fact that the two types of astrocytomas also present certain differences in their oncogenesis [14].

The aim of this review is to summarize the data on certain genetic mutations associated with the development and progression of astrocyt^14^as and gliomas of various degrees of malignancy, as well as the potential of using them for predicting and diagnosing this type of tumors, including SCA.

**Genetic markers associated with astrocytomas**

There is abundant evidence of the leading role played by genetic aberrations in the development and progression of primary malignant tumors of the CNS [17–20]. Such aberrations can include complete loss or partial deletion of the chromosome, loss of specific alleles, inactivating mutations, as well as methylation of the gene promoter. Next, we describe in detail some of the most crucial genetic markers associated with astrocytomas, as well as potential marker genes, and consider the prospects of their use for diagnostic and prognostic purposes.

*BRAF. *The *BRAF *gene, which encodes serine/threonine protein kinase of the RAF protein family, is a proto-oncogene involved in the regulation of cell proliferation and growth [21]. Mutations in this gene can lead to various tumors. For instance, duplication and activation of *BRAF *are found in juvenile PA, which is localized in the cerebellum (80%) and the hypothalamic/chiasmal region (62%) [22]. In some of the PA cases, a hybrid form of the *BRAF *gene has been found, which is formed by fusion with the previously uncharacterized *KIAA1549 *gene; this form is distinguished by constitutive activation of BRAF kinase [23, 24]. An activating point mutation, i.e. the substitution of valine to glutamate at position 600 (*BRAF *V600E) [25], as well as several other insertion mutations, are also known [26, 27]. Since this mutation is practically absent in other gliomas and non-glial tumors, it can be used for differential diagnosis and targeted therapy of PA [28]. However, it should be noted that, in some cases, mutations in *BRAF *can be found in diffuse gliomas and malignant astrocytomas, in combination with mutations in other genes, such as *CDKN2A *or *IDH *[29, 30]. According to a number of studies, the point mutation V600 in *BRAF *is more often found in supratentorial PA while hybrid oncogenes are mostly associated with PA located in the basicranial region and the spinal cord [31]. According to the multicenter study on SCA, more than 80% of PAs contain mutations in *BRAF*, with 40% of these cases being presented with a *BRAF*-KIAA1549 mutation and the remaining 60% being presented with *BRAF *duplication variants [32].

*CDKN2A. CDKN2A*, which encodes cyclin-dependent kinase that functions as a tumor suppressor, is another gene crucial to SCA and, in particular, PA [31]. In a cohort of 140 cases of PA, homozygous deletions in this gene were much more common in PAs localized in the brain stem and the spinal cord than in the case of PAs localized in the brain or cerebellum [33]. In addition to PA, deletions in *CDKN2A *are quite often detected in glioblastomas in adult patients. For instance, according to the results of two studies, this mutation was found in about half of the studied glioblastoma cases [34, 35]. In another study, a mutation in this gene was identified in three out of nine patients with high-grade glioblastomas of the spinal cord [36].

*IDH1/IDH2. *One of the most important discoveries in the study of gliomas (including astrocytomas) was the identification of mutations in the *IDH1 *and *IDH2 *genes encoding NADP^+^-dependent homodimers of isocitrate dehydrogenases 1 and 2, which are localized in the cytoplasm and mitochondria, respectively, and catalyze oxidative decarboxylation of isocitrate with the formation of α-ketoglutarate (α-KG) [37]. The *IDH1 *mutation is rarely found in primary glioblastomas ( < 5%). However, it is diagnosed in 70%–80% of grades II–III astrocytomas and secondary glioblastomas [38, 39]. The *IDH2 *mutation is much less common (less than 3% of all gliomas) and never found together with the *IDH1 *mutation [39]. In the overwhelming majority of cases (> 90%), the *IDH1 *mutation is presented with a substitution of arginine to histidine at position 132 (the enzyme active center). The mutant enzyme variant catalyzes the reduction of α-KG to 2-hydroxyglutarate (2-HG), a competitive inhibitor of α-KG-dependent dioxygenases, thus resulting in genome hypermethylation, which presumably occurs due to inhibition of the TET methylcytosine hydroxylase [40, 41]. In addition, these mutations can alter the histone methylation level by suppressing cell differentiation [42] and also contribute to the accumulation of the hypoxia-induced factor HIF-1α, which affects a number of processes, such as angiogenesis, cell metabolism, growth, differentiation, and apoptosis [43].

Tumors with mutations in *IDH *also typically carry a mutation in the *TP53 *gene or 1p/19q codeletion. These additional mutations are mutually exclusive; they are characteristic of astrocytomas (*TP53*) and oligodendro gliomas (1p/19q) [44]. The incidence of the *IDH1 *mutation in low-grade diffuse astrocytomas and secondary glioblastomas is 88% and 82%, respectively, with the *TP53 *mutation being detected in 63% of diffuse astrocytomas [44]. Only a few percents of cases with mutations in *IDH1 *or *IDH2 *were also characterized by changes in the *PTEN*, *EGFR*, *CDKN2A, *and *CDKN2B *genes. Meanwhile, the incidence of *TP53 *mutations was significantly lower (18%) in the samples carrying wild-type *IDH1 *and *IDH2*, while mutations in *PTEN*, *EGFR*, *CDKN2A*, and *CDKN2B *were much more frequent (74%). No cases of later occurrence of the *IDH1 *mutation after the *TP53 *mutation or codeletion were noted, which allows us to conclude that the *IDH1 *mutation appears at the earliest stages of oncogenesis and that it is possibly the common early event in the pathogenesis of gliomas of various histological variants.

*IDH *mutations have never been detected in PAs, which corresponds to the extremely rare transformation of PA into malignant tumors [44]. In addition, *IDH *mutations are very rarely found in primary glioblastomas [38]. This fact allows using *IDH1 *and *IDH2 *as markers for distinguishing between low-grade diffuse astrocytomas and secondary glioblastomas from PAs and primary glioblastomas.

According to some data, the frequency of *IDH1 *and *IDH2 *mutations in intracranial astrocytomas and glioblastomas is 68% and 12%, respectively [45]. Yet, there are no accurate data on the frequency of such mutations in SCA, which may be due to the rare incidence of this type of astrocytomas and the small sample size, which does not allow for a statistical analysis [3, 14]. For instance, the study focused on grades II and III SCA (*n *= 9) revealed no *IDH1 *R132H mutation, which is the most frequent mutation in intracranial astrocytomas [35]. Another multicenter study on SCA (*n *= 17) also demonstrated the absence of *IDH *mutations in the patients [32]. These results suggest the existence of potential genetic differences between intracranial and spinal tumors at the same histopathological stages.

*ATRX. *In addition to the accompanying *TP53 *and 1p/19q mutations, gliomas with mutations in *IDH *are distinguished by the presence of mutations in the *TERT *and *ATRX *genes, which are involved in telomere elongation. The *TERT *mutation correlates with the 1p/19q codeletion and primary glioblastomas; it is rarely detected in grade II and III astrocytomas and secondary glioblastomas [46]. The *ATRX *mutation is considered a hallmark of astrocytic tumors; it is closely associated with the *IDH *mutation in diffuse astrocytomas and secondary glioblastomas [47]. The *ATRX *mutation is quite rare in the absence of the *IDH *mutation [48]. In addition, *IDH *and *ATRX *mutations are very often associated with the *TP53 *mutation, which suggests a cooperative pathogenesis mechanism involving these three proteins [49].

The *ATRX *gene encodes the protein involved in DNA methylation and regulation of the expression of a number of genes. In addition, *ATRX *is associated with the ALT phenotype of tumors, which correlates with the emergence of telomeres of heterogeneous length in the cell; it also regulates the association of histone H3.3 with the telomeric DNA regions and a series of binding sites [50]. Mutations in *ATRX *lead to activity loss by its protein product, which causes typical developmental disorders, such as mental retardation, urogenital abnormalities and alpha-thalassemia. At the cellular level, these impairments manifest themselves by an altered DNA methylation pattern, failure of chromosome disjunction, and telomere dysfunction [51].

The incidence of the *ATRX *mutation in children diagnosed with glioma reaches 30% [52]. In adult patients, this mutation is noted in 71% of grade II–III astrocytomas and 57% of secondary glioblastomas, while in primary glioblastomas its incidence is only 4% of cases [48]. The *ATRX *mutation is found in pilocytic astrocytomas with anaplasia signs [53]. It should be noted that this mutation is more typical of young patients and can serve as a diagnostic and a prognostic factor, since it allows differentiation of astrocytomas and oligodendrogliomas and also because it is associated with a more benign prognosis (in case of lost ATRX activity) [54].

There are almost no data on the frequency of the *ATRX *mutation in SCA. A total of two cases have been reported describing such a mutation in grades II and III diffuse astrocytoma of the spinal cord [55, 56]. The summary data of the analysis of the two groups of patents (≤ 20 years and > 20 years) with high-grade spinal cord gliomas indicate an absence of this mutation in the younger group (*n *= 5) and its presence in 43% of older patients (*n *= 7) [57]. In addition, this mutation was also found in IDH-negative brain glioblastoma [57].

*H3F3A. *The *H3F3A *gene encodes the replication-independent histone H3.3, which participates in the structural organization of chromatin via active binding to transcription sites, as well as association with active and open chromatin [58]. Heterozygous mutations in the *H3F3A *gene are found in almost 80% of brainstem glioblastomas. Moreover, two mutually exclusive variants, namely substitution of lysine to methionine at position 27 (K27M) and substitution of glycine to arginine or valine at position 34 (G34R/V), are found in such cases [52, 59]. Both mutations are localized at positions close to the N terminus of the molecule, which undergoes a post-translational modification. Trimethylation of Lys27 is associated with decreased gene expression, while acetylation activates transcription. In addition, the methylation of Lys27 is crucial for a proper functioning of the PRC2 complex involved in transcription inhibition and cell differentiation [60, 61]. The mutations abrogate these modifications and processes, which, apparently, can trigger the onset of glioma.

Certain mutations in *H3F3A *are found in tumors of specific localization with a specific level of expression of OLIG1, OLIG2, and FOXG1 transcription factors. Gliomas with different mutations in *H3F3A *are believed to have different cellular origins [52, 62]. The G34R/V mutation is mainly found in children diagnosed with intracranial non-midline glioblastomas [52, 59]; the frequency of this mutation is 20%–30% [63]. The K27M mutation is mainly found in malignant astrocytomas of the thalamus, and brainstem and the spinal cord are prevalent in adolescents and children [57, 64]. The K27M mutation is associated with high tumor aggressivity, even if it is classified histologically as low-grade astrocytoma [65]. However, according to some data, the prognosis of thalamic gliomas in adults carrying this mutation may not appear worse than that in patients without the aberration, which suggests heterogeneity of this molecular subgroup of diffuse gliomas [66].

The K27M mutation is often associated with mutations in *TP53 *(thalamic gliomas) and chromosome 10 monosomy, while it is rarely diagnosed together with mutations in *BRAF *(V600E) and *ATRX *and never found together with mutations in *IDH1 *and *EGFR *[64, 66, 67]. This incompatibility with *IDH1 *is due to the fact that the mutation makes Lys27 methylation possible [62, 68]. Schwartzentruber et al. [52] demonstrated that the *ATRX *mutation is much more frequently associated with the G34R/V mutation than with the K27M mutation in *H3F3A*.

The K27M mutation in *H3F3A *in patients with spinal cord astrocytomas is associated with grade III and IV tumors. This mutation was detected in 61% of patients older than 20 years (*n *= 18) and in 54% of patients younger than 19 years (*n *= 24) diagnosed with grade III–IV SCA [57]. In another study, this mutation was found in 28% (*n *= 32) of patients with SCA but the malignancy grade of the astrocytomas with a confirmed mutation was not indicated [69]. Johnson et al. [36] revealed the K27M mutation in 77.8% of cases (*n *= 9) of spinal cord glioblastomas. Another study conducted in a cohort of 36 primary diffuse gliomas of the spinal cord showed approximately the same mutation frequency rate for grade III–IV gliomas in adults and children (52% and 54%; *n *= 11 and 19, respectively) [70]. Thus, this mutation is quite often associated with grade III–IV spinal cord gliomas. It should be noted that K27M is not present in other types of malignant tumors [71] and, therefore, may be pathognomonic for the primary spinal glioblastoma and may also serve as an indicator of the worst prognosis [64].

*TP53. *Protein P53 is a transcription factor that regulates the transcription of the thousands of genes involved in the cell cycle, cell differentiation, and apoptosis. Mutations in *TP53 *are among the earliest genetic changes in tumor cells and are found in 60% of the precursor cells of low-grade astrocytomas [72]. These mutations are present in most secondary glioblastomas (65%), mainly in codons 248 and 273. In primary glioblastomas, mutations in various codons of *TP53 *were found in 30% of patients [73].

Mutations in *TP53 *provoke a more aggressive growth of grade I–II astrocytomas: i.e., they are considered an unfavorable prognostic factor [74]. As in the case of *ATRX*, the mutation in *TP53 *is mutually exclusive with the 1p/19q codeletion typical of oligodendrogliomas. Detection of this mutation can serve as proof of a diagnosis of astrocytoma [75]. It is an interesting fact that, in contrast to intracranial glioblastomas, a *TP53 *mutation in spinal cord glioblastomas is often detected in the absence of a *IDH1 *mutation [14].

*TP53 *mutation is often found in grade III–IV SCA. For instance, Govindan et al. [76] revealed the mutation in five out of six glioblastomas, while Walker et al. [77] reported the presence of the mutation in 60% of diffuse astrocytomas. Similar data were obtained by Johnson et al. [36] for patients with high-grade spinal cord glioblastomas (66.7%). Overexpression of *P53 *was diagnosed in 57% of patients over 20 years of age (*n *= 7) with grade III–IV spinal cord glioblastomas and in 40% of patients younger than 20 years of age (*n *= 5) [57].

*PTEN. *The *PTEN *gene encodes phosphatase PTEN and belongs to tumor suppressor genes. Phosphatase PTEN is involved in dephosphorylation of the membrane-bound phosphatidylserine PIP3 to PIP2, which regulates the PKB/AKT signaling pathway. In case of gene loss or its mutation, its function cannot be performed by other enzymes [78]. Impaired expression of *PTEN *results in constitutive activation of the PKB/AKT pathway, which, in turn, triggers a series of processes associated with the cell cycle, cell proliferation, migration, and angiogenesis. PTEN also regulates the mTOR signaling pathway, which controls the self-renewal and differentiation of tumor stem cells. Deletion in the *PTEN *gene increases the size of these cells and causes their proliferation rate to increase and the suppression of the apoptosis of neural progenitor cells [79]. Atypical migration of progenitor cells carrying a *PTEN *mutation can lead to cerebellar and hippocampal dysplasia, followed by gliomagenesis. However, additional mutations, for instance, mutations in *TP53*, are required for the initiation of neoplastic changes [80]. Deletions in chromosome 10 in the region of *PTEN *are often found in tumors characterized by *EGFR *amplification [72]. However, mutations in this gene, on the contrary, are poorly associated with EGFR [81].

Inactivation of *PTEN *usually caused by an inactivating point mutation (12%) or deletion of the long arm of the 10q chromosome (32%) [82] occurs in various types of tumors, including astrocytomas. In the latter case, *PTEN *mutations are extremely rarely found in PA but are present in 18% of anaplastic astrocytomas and up to 40% of glioblastomas, mainly the primary ones [31, 82, 83]. Rare detection of *PTEN *mutations in grade I–II astrocytomas and secondary glioblastomas may be associated with methylation of the *PTEN *promoter, which is often found in low-grade gliomas and reduces PTEN protein production compared to the normal level [84]. Mutations in the *PTEN *gene are more common among older patients with anaplastic astrocytoma and young patients with glioblastoma [83]. Only sporadic reports of *PTEN *mutations in such a rare tumor as grades III and IV SCA are known [56].

From the prognostic point of view, a loss of the PTEN function is associated with higher tumor aggression and decreased survival of patients with anaplastic astrocytoma, whereas no correlations were found for glioblastoma [12].

*EGFR. *The *EGFR *gene encodes the epidermal growth factor receptor. EGFR is a transmembrane glycoprotein consisting of an extracellular ligand-binding domain, a hydrophobic transmembrane domain, and a cytoplasmic tyrosine kinase domain. Binding of a ligand by EGFR results in dimerization and autophosphorylation of the receptor, as well as phosphorylation of cell substrates, which triggers a cascade of intracellular receptors associated with cell division and proliferation.

Increased expression or amplification of the *EGFR *gene is characteristic of many tumors. In addition to overexpression and amplification, point mutations and structural rearrangements can also occur in the gene, thus altering the functional characteristics of its product. *EGFR *nucleotide sequences corresponding to its extracellular and intracellular domains hold certain positions that are most susceptible to mutagenesis [85]. Most *EGFR *mutations in gliomas, including EGFRvIII, affect the extracellular domain of the receptor, while being mainly associated with the intracellular domain in non-glioma tumors [86, 87]. About half of glioblastomas with *EGFR *amplification also contain deletions in exons 2–7. The product of EGFRvIII mutation is a constitutively active EGFR variant stimulating tumor angiogenesis in malignant gliomas [88]. As an activator of cell proliferation, EGFRvIII is expressed only by a specific fraction of glioblastoma cells, thus inducing proliferation not only of these cells, but also of the adjacent cells expressing wild-type EGFR [89].

Mutations in *EGFR *and *TP53 *are mutually exclusive in glioblastomas [90]. As in the case of *PTEN *mutations, a mutation in *EGFR *is typical of primary glioblastomas; it is rare in secondary ones [91]. Overexpression of the gene was revealed in 60% of primary glioblastomas, while the remaining 40% carried the amplified gene. In addition, overexpression or amplification of *EGFR *was found in 33% of patients with anaplastic astrocytomas and in less than 10% of patients with oligodendrogliomas [85]. It is also known that changes caused by *EGFR *gene aberrations appear only in 3% of astrocytomas and glioblastomas carrying *IDH *mutations, while the frequency of such changes is much higher in the presence of wild-type IDH [35, 37].

SCA is a rare tumor. Thus, there is not enough data regarding the incidence of this marker to make any statistical inferences. Two cases of EGFR-positive anaplastic astrocytoma have been reported by Korean researchers [92, 93]. Another two studies mentioned EGFR-positive spinal cord glioblastomas. The marker was found in two out of six cases [76] and in three out of nine cases [56] in those studies, respectively.

Amplification and overexpression of *EGFR *is considered to be associated with a high degree of glioma malignancy, aneuploidy, and proliferative index, while a mutation in EGFRvIII is potentially associated with an aggressive disease course, refractoriness to therapy, and poor prognosis [94, 95]. Moreover, overexpression of *EGFR *significantly decreases the chances of survival of patients with anaplastic astrocytomas [96], which allows one to ascribe them to the subgroup with a poor prognosis [97].

**Practical significance of the molecular markers associated with astrocytomas**

To date, the histomorphological classification of tumors serves as the basis for predicting the course of oncological diseases. However, such a diagnosis based on visual evaluation criteria is to some extent subjective, sometimes leading to significant discrepancies in the evaluation of histological specimens. In addition, the clinical course of the disease in some cases is poorly correlated with the histomorphological classification, while tumors with a similar histological characterization may respond differently to the same therapy. In this regard, the interest in molecular markers as means for a more accurate disease classification and prognosis has increased in recent years.

**Fig. 3:**
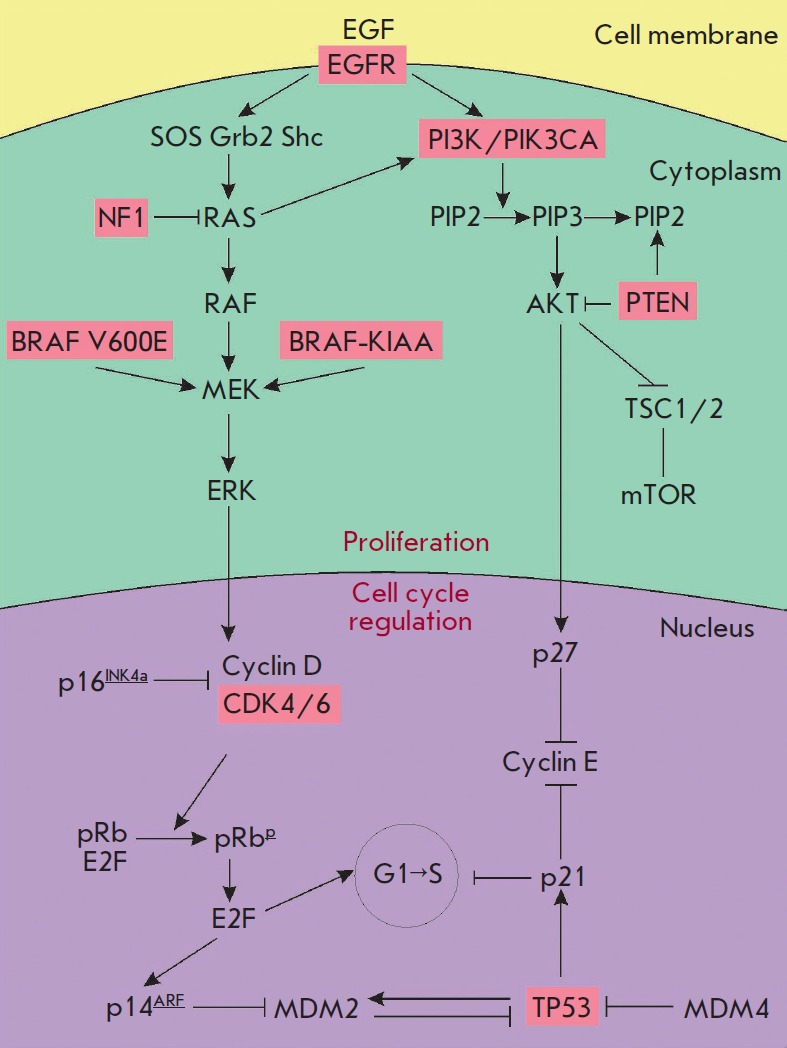
Simplified scheme of the signaling pathways associated with the pathogenesis of glial tumors and the impact of the mutations associated with astrocytomas. The data are presented according to [99], with modifications

A vast number of studies conducted over the last 10–15 years have significantly improved our under standing of the mechanisms of the onset and progression of CNS glial tumors and revealed the key genes whose mutations or aberration can be considered potential prognostic and diagnostic factors (*Fig. 3*). In 2016, a series of molecular markers were included into the WHO Classification of CNS tumors. For instance, the *IDH *mutation test has become a part of the routine diagnosis and classification of gliomas [14].

**Table 1:** Structures of K_V_-channels alone and in complex with charybdotoxin used in homology modeling studies

Gene/ mutation	Mutation frequency in astrocytomas				Annotation
	PA	DA	AA	GB (prim. and sec.)	
**BRAFKIAA1549**	32%	Rare			It is more common for PA localized in the spinal cord and in the basilar region.
**BRAF V600E**	48%	Rare			It can be used for PA differentiation; it is most frequently found in supratentorial PA. It serves as a positive prognostic marker in children and young patients.
IDH1	-	< 70%		70–80% (sec.) < 5% (prim.)	IDH1 and IDH2 are mutually exclusive. mutIDH: positive prognostic marker wtIDH: more aggressive course. IDH1: possible application for exclusion of PA and GB1.
IDH2	-	< 3%			
**ТР53**	-	29% (increased expression)		65% (sec.) 30% (prim.)	More aggressive disease course. Mutually exclusive to the 1p/19q codeletion; can be potentially used for astrocytoma differentiation. **Revealed in 60%–67% of grades III–IV SCA.**
**ATRX**	+	60–70%		57% (sec.) 4% (prim.)	It rarely appears in the absence of mutations in IDH and ТР53, it is mutually exclusive to 1p/19q codeletion. It can be used for differentiation of astrocytomas and 1p/19q codeletion. The prognosis is more favorable in case of a loss of th ATRX activity.
**H3F3A** **K27M**		+		+ (50% in prim. spinal cord GB)	Mostly present in children. Midline tumors of the brain and the spinal cord. Never diagnosed together with IDH1 and EGFR. Often found together with ТР53. **Apparently, pathognomonic for primary GB of the spinal cord.**
H3F3A G34R/V				20–30%	Present in adolescents and young patients. More favorable prognosis. Non-midline intracranial glioblastomas. Often found together with the ATRX, TP53, and PDGFRA mutations.
**EGFR**	-	+	33%	100% (prim.) rare (sec.)	Typical for primary GB. Rarely found together with the mutation in IDH, mutually exclusive to ТР53 mutation. Associated with high malignancy and poor prognosis.
FGFR2	+	3.5%		2.5%	Mutually exclusive mutations in IDH and EGFR. The expression level decreases increasing the malignancy degree.
**PDGFRA**	-	3–69%	12–33%	31% (mainly sec.)	
**PTEN**	Extremely rare	Rare	18%	40% (mainly prim.)	More aggressive course in case of anaplastic astrocytomas.
**NF1**	15–20%	+	+	15–18% (prim.)	Is associated mainly with astrocytomas.
**CDKN2A**	+		+	+	

Note. Mutations found in astrocytomas of the brain and spinal cord are shown in bold. The symbols + and – stand for the presence or absence of a mutation in the specific type of astrocytoma; an empty cell means a lack of information. The presented data are based on information reviewed in the current paper.

Since the number of studies related to SCA-associated genetic changes is substantially lower than that of the studies devoted to brain astrocytomas, the current review considers markers of brain gliomas, including both the well-studied and those that are still under assessment for potential use. General information on the detection frequency of the 16 markers examined in various types of astrocytomas, their features, and prognostic value is presented in *Table*.

**Fig. 4:**
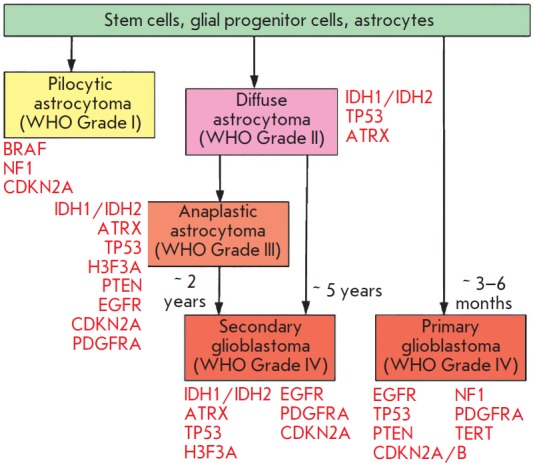
The most common genetic changes associated with the development of astrocytomas of various degrees of malignancy. The data are adapted from [100], with modifications

The information accumulated to date allows us to draw certain conclusions and make assumptions about the association of specific mutations with various types of astrocytomas (*Fig. 4*), a patient’s age, other mutations, as well as a possible disease prognosis. For example:

– pilocytic astrocytomas mainly contain mutations in the *BRAF*, *NF1 *and *CDKN2A *genes;

– mutations in *IDH1*, *ATRX *and *TP53 *are mainly associated with primary glioblastomas and grade II–III astrocytomas (often found in combination with each other);

– mutations in *H3F3A *are mainly diagnosed in grades III–IV astrocytomas and, apparently, (in the case of a K27M mutation) are pathognomonic for the primary spinal glioblastomas;

– mutations in *EGFR *and *PTEN *are mostly associated with primary glioblastoma as well as anaplastic astrocytomas; and

– a mutation in *PDGFRA *is predominantly found in secondary but not primary glioblastomas.

The mutation V600E in *BRAF *(in children and adolescents) serves as a positive prognostic marker of grades I and II astrocytomas [98]. *H3F3A *K27M, *TP53*, *EGFR*, and *PTEN *are mutations that worsen the disease course and the overall prognosis.

Mutations in *IDH *are a crucial prognostic feature which allows one to divide diffuse infiltrative gliomas into three groups [99]. The most favorable prognosis is characteristic of the combination of mutant *IDH *(mutIDH) and the 1p/19q codeletion. The worst disease course is characteristic of tumors carrying wild-type *IDH *(wtIDH). Such tumors are usually aggressive and similar to primary glioblastomas in their molecular characteristics (aberrations in *EGFR, PTEN, NF1, CDKN2A/B*). The third group, for which the prognosis turned out to be intermediate between the two, includes mutIDH in the absence of 1p/19q codeletion. In the overwhelming majority of cases, this variant is associated with mutations in *TP53 *and *ATRX*. Regardless of the malignancy degree and histological characteristics of the tumor, the prognosis for this variant is always more favorable than that for wtIDH.

It should be noted that the molecular profiles of astrocytomas in children differ significantly from the adult variants and mainly contain mutations in such genes as *BRAF*, *H3F3A*, and *ATRX *[99].

To date, there is no information on any identification of markers such as *IDH1/2, H3F3A *G34R/V, and *FGFR2 *in SCA. Pilocytic astrocytomas of the spinal cord were shown to be associated with mutations in the *BRAF*, *CDKN2*, *NF1*, and *PTEN *genes, while malignant grades III–IV SCA variants are associated primarily with *H3F3A *K27M (mostly young patients and children), *TP53*, and *PTEN *[32]. The remaining mutations discussed in the current review have been reported mainly as sporadic cases and cannot be used to make any statistical inferences.

In addition to their prognostic and diagnostic values, biomarkers can also be used in the development of drugs for targeted therapy of astrocytomas. For example, partial efficacy of selective inhibitors of isocitrate dehydrogenase with the *IDH1 *R132H mutation has been shown both *in vitro *and in glioma models [100]. Preliminary tests of the JNJ-42756493 drug *in vitro *and *in vivo *confirmed that growth of a tumor carrying recombinant *FGFR-TACC *was inhibited in two patients in whom the standard therapy had earlier been ineffective [101]. Some targeted drugs, such as MAb- 425 and nimotuzumab (targeted against EGFR), as well as crenolanib and nilotinib (targeted against PDGFR), are already in phases II–III of clinical trials [102]. At the same time, it is necessary to understand that the drugs that have shown good results in the treatment of intracranial astrocytomas may turn out to be ineffective against SCA, due to the possible differences in their genetic profiles.

Currently, not all molecular markers associated with astrocytomas (especially with the even less common SCA type) show potential for clinical usage, taking into account their prognostic, diagnostic, or therapeutic value. In some cases, this is due to insufficient information on the detected genetic aberrations. Recently, retrospective studies of clinical tissue samples aimed at identifying target molecular markers have been carried out. Such studies allow researchers to cover up to several hundred samples and obtain statistically significant genetic landscapes of target tumor types. Further research in this direction can provide much better elucidation of the genetic and epigenetic changes that occurr in tumor cells, it can help identify new promising biomarkers, and develop innovative strategies for the diagnosis and treatment of astrocytomas.

## Abbreviations

CNS: central nervous system
IMSCT: intramedullary spinal cord tumors
PA: pilocytic astrocytoma
SCA: spinal cord astrocytoma
DA: diffuse astrocytoma
AA: anaplastic astrocytoma
GB: glioblastoma;
WHO: World Health Organization
